# Chromosomal alterations in the clonal evolution to the metastatic stage ofquamous cell carcinomas of the lung

**DOI:** 10.1054/bjoc.1999.0878

**Published:** 1999-12-08

**Authors:** S Petersen, M Aninat-Meyer, K Schlüns, K Gellert, M Dietel, I Petersen

**Affiliations:** Institute of Pathology, Department of Surgery, University Hospital Charité Schumannstr 20–21, Berlin, D–10098 Germany

**Keywords:** lung cancer, tumor progression, CGH

## Abstract

Comparative genomic hybridization (CGH) was applied to squamous cellcarcinomas (SCC) of the lung to define chromosomal imbalances that are associated with the metastatic phenotype. In total, 64 lung SCC from 50 patients were investigated, 25 each with or without evidence of metastasis formation. The chromosomal imbalances summarized by a CGH histogram of the 50 cases revealed deletions most frequently on chromosomes 1p21–p31, 2q34–q36, 3p, 4p, 4q, 5q, 6q14–q24, 8p, 9p, 10q, 11p12–p14, 13q13–qter, 18q12–qter and 21q21. DNA over-representations were most pronounced for chromosomes 1q11–q25, 1q32–q41, 3q, 5p, 8q22–qter, 11q13, 12p, 17q21–q22, 17q24–q25, 19, 20q and 22q. In ten cases, paired samples of primaries and at least one metastasis were analysed. The comparison revealed a considerable chromosomal instability and genetic heterogeneity; however, the CGH pattern indicated a clonal relationship in each case. The difference in histograms from the metastatic and non-metastatic tumour groups was most useful in pinpointing chromosomal imbalances associated with the metastatic phenotype, indicating that the deletions at 3p12–p14, 3p21, 4p15–p16, 6q24–qter, 8p22–p23, 10q21–qter and 21q22, as well as the over-representations at 1q21–q25, 8q, 9q34, 14q12 and 15q12–q15, occurred significantly more often in the metastatic tumour group. The comparison of the paired samples confirmed these findings in individual cases and suggested distinct genetic changes, in particular the extension of small interstitial deletions, during tumour progression. Importantly, metastasis-associated lesions were frequently detectable in the primary tumour providing a method of identifying patients at risk for tumour dissemination. Individual profiles and histograms are accessible at our web site http://amba.charite.de/cgh. © 2000 Cancer Research Campaign


*British Journal of Cancer* (2000) **82**(1), 65–73
© 2000 Cancer Research Campaign
Article no. bjoc.1999.0878

### Chromosomal alterations in the clonal evolution to the metastatic stage of squamous cell carcinomas of the lung

**S Petersen[1], M Aninat-Meyer[1], K Schlüns[1], K Gellert[2,*], M Dietel[1] and I Petersen[1]**

[1]Institute of Pathology and [2]Department of Surgery, University Hospital Charité, Schumannstr 20–21, D-10098 Berlin, Germany

**Summary** Comparative genomic hybridization (CGH) was applied to squamous cell carcinomas (SCC) of the lung to define chromosomal imbalances that are associated with the metastatic phenotype. In total, 64 lung SCC from 50 patients were investigated, 25 each with or without evidence of metastasis formation. The chromosomal imbalances summarized by a CGH histogram of the 50 cases revealed deletions most frequently on chromosomes 1p21–p31, 2q34–q36, 3p, 4p, 4q, 5q, 6q14–q24, 8p, 9p, 10q, 11p12–p14, 13q13–qter, 18q12–qter and 21q21. DNA over-representations were most pronounced for chromosomes 1q11–q25, 1q32–q41, 3q, 5p, 8q22–qter, 11q13, 12p, 17q21–q22, 17q24–q25, 19, 20q and 22q. In ten cases, paired samples of primaries and at least one metastasis were analysed. The comparison revealed a considerable chromosomal instability and genetic heterogeneity; however, the CGH pattern indicated a clonal relationship in each case. The difference in histograms from the metastatic and non-metastatic tumour groups was most useful in pinpointing chromosomal imbalances associated with the metastatic phenotype, indicating that the deletions at 3p12–p14, 3p21, 4p15–p16, 6q24–qter, 8p22–p23, 10q21–qter and 21q22, as well as the over-representations at 1q21–q25, 8q, 9q34, 14q12 and 15q12–q15, occurred significantly more often in the metastatic tumour group. The comparison of the paired samples confirmed these findings in individual cases and suggested distinct genetic changes, in particular the extension of small interstitial deletions, during tumour progression. Importantly, metastasis-associated lesions were frequently detectable in the primary tumour providing a method of identifying patients at risk for tumour dissemination. Individual profiles and histograms are accessible at our web site *http://amba.charite.de/cgh*. © 2000 Cancer Research Campaign

**Keywords:** lung cancer; tumor progression; CGH

Lung cancer carries the highest cancer-related mortality throughout the world and has an incidence of about 60 new cases per year in 100 000 people (Pisani et al, 1993). The major distinction of clinical importance is made between small-cell lung cancer (SCLC) (~20%) and non-small-cell lung cancer (NSCLC) (~80%). The histopathological diagnosis of SCLC is highly predictable for the clinical course since almost every tumour has metastasized at the time of diagnosis, or will do so within the near future. In contrast, it is far more difficult to predict the outcome of NSCLC based on morphological grounds. Some tumours are similar to SCLC with a highly aggressive behaviour and early metastasis formation, whereas others may be cured after surgery or remain stable for a considerable period of time even in the case of residual disease.

Squamous cell carcinomas (SCCs) together with adenocarcinomas constitute the two major subtypes of NSCLC. Adenocarcinomas are typically located in the lung periphery and often cause first symptoms by a distant metastasis. In contrast, SCCs develop preferentially from the bronchial epithelium of the central lung and may present regional lymph node metastases prior to haematogeneous spread. However, the distribution of blood-borne metastases is similar in both entities affecting almost any organ, but with preference to the liver, bone, adrenal gland and the nervous system.

*Received 5 January 1999*
*Revised 1 June 1999*
*Accepted 8 June 1999*

*Correspondence to:* I Petersen. E-mail: iver.petersen@charite.de

The genetic mechanisms underlying lung cancer development and progression are only beginning to emerge. Many defects have only been described on the chromosomal level and, although several genetic lesions have been identified, many still remain unknown. The chromosome regions identified by allelotyping suggesting the involvement of tumour suppressor genes include 1p, 2q, 3p, 4p, 4q, 5q, 6p, 6q, 8p, 9p, 10q, 11p, 11q, 14q, 17q, 18q and 22q (Sekido et al, 1998). Comparative genomic hybridization (CGH) also detected deletions at 1p, 2q, 3p, 4p, 4q, 5q, 6q, 8p, 9p, 10q, 11p, 11q, 13q, 17p, 18p, 18q and 21q, as well as over-representations on 1q, 3q, 5p, 8q, 11q13, 12p, 17q, 19q, 20q and 22q (Ried et al, 1994; Levin et al, 1994, 1995; Petersen et al, 1997*a*, 1997*b*; Schwendel et al, 1997).

We recently took advantage of the fact that the primary data in CGH are derived from digital analysis of fluorescence images and are already computerized to calculate histograms and difference histograms for statistical analysis of tumour groups (Petersen et al, 1997*b*). In the present study we applied this approach to evaluate the incidence of chromosomal alterations in SCC and to highlight chromosomal subregions being associated with the metastatic phenotype. The statistical analysis of the tumour subgroups was supplemented by the direct comparison of primary and metastatic tumours in ten cases.

*Present address: Department of Surgery, Oskar-Ziethen-Hospital, Berlin-Lichtenberg, Germany

### MATERIALS AND METHODS

### Tumour samples

Tumour specimens were mainly obtained from surgical resections at the Department of Surgery of the Charité Hospital at the Humboldt University Berlin. Operation specimens were transferred to the Institute of Pathology within 1 h after surgical removal. Generally, no adjuvant radiotherapy or chemotherapy was applied before surgery. Additionally, tumour specimens of primary and metastatic lesions were collected at post-mortem examinations at the Pathology Institutes of the Charité, the University Hospital of Kiel and the University Hospital of Zurich. These specimens were frozen 3–48 h after the patients died. One aliquot was frozen in liquid nitrogen and kept at –80°C until DNA extraction. DNA was extracted from several 30 μm cryostat tissue sections by proteinase K and phenol–chloroform extraction which was verified to consist of a minimum of 70% tumour cells in each case. A second aliquot was submitted to formalin fixation and paraffin embedding. The histopathological diagnosis was established in every case according to the WHO guidelines on haematoxylin and eosin (H&E)-stained tissue sections (WHO, 1981).

Samples from 50 patients with SCC were investigated. One group consisted of 25 non-metastatic (pN0/pM0) tumours that were derived from surgical resections, except one autopsy case (L101, no information on treatment). In the second group, 19 primary and 20 metastatic lesions (18 haematogeneous and two lymph node metastases) from another 25 patients, seven with stage pN2 and 18 with stage pM1 disease, were investigated. Of these, 15 cases were autopsy cases (radiotherapy in six cases: L24, L26, L29, L30, L34, L36; no treatment in seven cases: L23, L27, L33, L35, L37, L39, L40; no information on treatment in two cases: L28, L31) and ten resections. Paired samples of primary tumour and corresponding metastases were analysed in ten cases (seven autopsy cases: L23, L27, L31, L34, L37, L39, L40; three resections: L171, L219, L220).

### Comparative genomic hybridization

Chromosome metaphase spreads, DNA labelling, hybridization and detection were performed as described previously (Petersen et al, 1997*a*, 1997*b*). Briefly, tumour and normal DNA were differentially labelled with biotin-16–dUTP and digoxigenin-11–dUTP (both Boehringer Mannheim, Germany) respectively. One microgram of each tumour and normal DNA, together with 30 μg human Cot1 DNA and 10 μg salmon sperm DNA were ethanol precipitated and applied to denatured and dried normal metaphase spreads. After 3 days of hybridization at 37°C, the tumour and normal genomes were specifically detected by avidin–FITC (fluorescein isothiocyanate; Vector Laboratories) and anti-digoxigenin–rhodamin–TRITC (Boehringer Mannheim, Germany) respectively. The chromosomes were counterstained with 4,6-diamino-2-phenolindol dihydrochloride (DAPI) and embedded in 90% glycerol containing 2.3% 4,6-diazabicyclo[2.2.2]octane (Sigma Aldrichs).

### Digital image analysis

Fluorescence images were obtained with a Zeiss Axiophot epifluorescence microscope in conjunction with a 12-bit cooled charge-coupled device camera (Photometrics, Tucson, AZ, USA). Three images per metaphase spread were acquired, quantitated as 8-bit grey level images and stored in a TIFF-format. The DAPI image was used for chromosome identification. FITC and TRITC, specific for the tumour and reference genome, respectively, were used to compute fluorescence ratio images and ratio profiles. Generally, 15 metaphases per karyogram were analysed for each case. CGH sum-karyograms as well as mean ratio profiles with confidence intervals (CI) were calculated (Petersen et al, 1997*a*; Roth et al, 1997).

### Statistical determination of chromosomal imbalances

Alterations were determined by calculating the mean FITC–TRITC profile with its 95% and 99% CIs. The profile was tested for significant deviations from the normal ratio of 1.0 by a Student's *t*-test (Bockmühl et al, 1997; Petersen et al, 1997*b*). This type of evaluation constitutes a quite sensitive measure for evaluation of DNA imbalances which is best explained by the example of Figure 2B. In case L37, the ratio profile of the primary tumour on the left showed only minute deviations from the normal ratio value of 1.0 represented by the middle line. The left and right lines represent the 0.75 and 1.25 ratio respectively. If these fixed thresholds, or even the less restrictive 0.8 or 1.2 thresholds were applied for the determination, no DNA imbalance would be scored. The statistical method, however, already counts those deviations in which the profile together with its CI is on one side of the 1.0 ratio line, e.g. the deletion at 10q23–q25.

We used the fixed ratio values 1.5 and 0.5 as thresholds to define pronounced DNA gains and losses respectively. The 1.5 ratio threshold has been already used by us to define high copy amplifications in SCLC (Petersen et al, 1997*a*). Similarly, pronounced DNA losses probably correspond to multicopy deletions within the tumour genome.

After the statistical determination of the chromosomal imbalances of each tumour, a histogram of all cases was calculated (Figure 1A). It includes one tumour sample per case and represents the incidence of DNA gains and losses of the tumour group along each chromosome, e.g. the maximum value of 100% is reached if all tumours of the same tumour group carry a change at a specific chromosomal region. The two significance levels are indicated by distinct colours: blue areas indicate those alterations with 99% significance, whereas the green areas include those with 95% significance. In addition, pronounced DNA copy number changes are depicted in red.

### Statistical comparison of tumour subgroups

The alterations with 99% significance were included into the difference histogram of Figure 1B. It represents the genetic imbalances of non-metastatic and metastatic SCC providing a statistical comparison of the two subgroups. Again, one tumour per case was included into the histogram, with preference for metastatic lesions in the pM1/pN2 subgroup. The primary tumour was used in ten cases (L24, L36, L33, L41, L42, L44, L143, L157, L172, L245), in which no metastasis was available. The percentage of changes occurring only in the pN0/M0 tumours is represented by the green colour, whereas the excess of changes in the metastatic tumour group is shown in red. The white areas beneath the coloured part of the histogram represent the percentage of changes that are present in both subgroups. A large coloured area thus indicates a

© 2000 Cancer Research Campaign

**Table 1** Comparison of genetic abnormalities between primary and corresponding metastatic tumours

| Case | Common changes | Different changes |
|---|---|---|
| L220 | 1p↓, 2p↑, 3pter-q13.3↓, 3q23-qter↑↑, 4pter-q26↓, 4q31↓, 5p↑↑, 5q↓,6p21-pter↓, 6q25-qter↓, 7q35-qter↓, 8p21-p23↓, 8q24↑, 9p11-p13↓, 9q↑, 10p14-pter↓, 10q21-qter↓, 11p↓, 11q13↑, 12q14-qter↓, 13q31-qter↓, 14q↑, 15q↓, 16q11-q21↑, 16q23-qter↓, 17q12-q24↑, 18q12-q22↓, 19p12-q13.3↑, 20p↑↑, 21q↓, 22q13↓ | 1q11-q25↑↑(ln), 1q↑(pt), 1q43-qter↓(ln), 4q27-q28↓(ln), 4q32-qter↓(ln), 6p11↑(pt), 6q15-q21↓(pt), 7pter-q31↑(pt), 7q32-q34↓(ln), 8q24↑↑(pt), 8q12-q23↑(pt), 8q21-q23↑(ln), 9p21-p22↓(ln), 10p12-p13↓(pt), 11q21-q23↑(ln), 12p11-p12↑(pt), 13q11-q22↓(ln), 18pter-q11↓(pt), 18q23↓(pt) |
| L219 | 1q↓, 2q12↑, 2q14-qter↓, 3pter-q25↓, 5q↓, 6q21-qter↓, 7p↓, 7q35-qter↓, 8p12-pter↓, 8q↑, 9↓, 10p15↓, 10q24-qter↓, 13q↓, 14q21↓, 15q21-qter↓, 18p↓, 18q11-q12↑, 19q13↑, 20q11-q12↑, 21q21-q22↓, 22q11-q12↑↑ | 1p32-pter↓(pt), 1p13-p31↓(ln), 4↓(ln), 5p↑(pt), 6p22-pter↓(ln), 6q11-q16↓(ln), 7q21-q34↓(ln), 10p11-p14↓(ln), 10q21↓(ln), 10q22↑(pt), 11p15↓(pt), 11p11-p14↓(ln), 11q12-q13↑(ln), 12p↓(ln), 12q15-q22↓(ln), 14q11.2↑(ln), 14q12-q13↓(pt), 14q22-qter↓(pt), 15q11-q12↑(ln), 16↓(pt), 17↓(pt), 17p11-p21↑(b), 18q21-qter↑(pt), 20p12-pter↓(ln), 22q22↓(pt) |
| L171 | 1p21-p31↓, 1q21-q24↑, 2q11-q12↑, 2q34-q36↓, 3p12-p14↓, 3p24-pter↓, 3q25-qter↑↑, 4q21-qter↓, 5q14-q23↓, 6p11↑, 6q14-q24↓, 7q11.2↑, 8p12-q22↓, 8q12-q21.3↓, 8q24-qter↑, 9p12-p21↓, 10q21-q22↓, 11q13↑, 12q13-q14↑, 12q24↑, 14q13-q22↓, 15q11-q12↑, 16p13↑, 17q12-q24↑, 18q12-q22↓, 21q11-q22↓, 22q11-q13↑ | 1p33-p34↑(lu), 1q25-qter↑(pt), 2p13-pter↑(lu), 2q21-q23↓(pt), 2q31-q32↑(lu), 3p21-p23↓(lu), 4p11-p15↓(lu), 4q16↑(pt), 5q12-q13↓(lu), 5q31-qter↓(lu), 7p21-pter↑(pt), 7q21-qter↑(lu), 7q31-q34↓(pt), 8q23-q24.1↑(lu), 9q21-q33↓(lu), 9q34↑(pt), 10p14-q21↓(lu), 10q23-q25↓(lu), 11p13-p14↓(pt), 11q14-q22↓(pt), 11q23-qter↓(lu), 12pter-q12↑(lu), 13q14-q33↓(pt), 16p11-p12↑(lu), 19pter-q13.2↑(pt) |
| L31 | 1p↓, 1q↑, 2q14↑, 2q37↑, 3p↓, 3q↑↑, 4q13-qter↓, 5p↑↑, 5q13-qter↓, 6p↓, 8↑↑, 9q↑, 10q11↓, 11q24-q25↓, 12pter-q12↑, 13q12↓, 14q↑, 15q11-q24↓, 16↑, 17↑, 18q↓, 20q↑, 21q↓, 22q11.2-q13↑ | 2p24-q13↑(pt), 2q21-q36↑(pt), 4p15-q12↓(ki), 6q↓(ki), 6q14-q24↑(pt), 7p↓(ki), 7q21-qter↓(ki), 10p↓(ki), 10q21-q25↓(ki), 11p14-p15↑(ki), 11q14-q23↓(ki), 12q14-23↓(ki), 12q13-q21↑(pt), 13q13-qter↓(ki), 19↓(pt), 19p↓(pt), 19↑ (ki) |
| L37 | 1p11-q2↑↑, 1q25-qter↑, 2q34-q37↓, 3p12-p23↓, 3q23-qter↑, 4p11-p15↓, 4q21-qter↓, 5q12-qter↓, 7q11↑, 8p12-pter↓, 8q22-qter↑, 9p11-pter↓, 9q11-q33↓, 10q11↑, 10q23-q25↓, 11p11-p15↓, 11q14-qter↓, 12pter-q12↑↑, 12q13-q15↑, 12q24-qter↑, 13q21-qter↓, 14q12-q31↓, 15q12-q21↓, 17p11-q11↑, 18q11↑, 19↑, 20q11-q12↑↑, 21q21q↓, 22q↑ | 1p33-pter↑(li), 2pter-q31↑(li), 3p24-pter↓(li), 5p11↑(pt), 5p15↑(pt), 6p12↑(li), 6p22-pter↓(li), 6q22↓(pt), 6p25–q26↑(li), 7p11-pter↑(li), 7q21-qter↑(li), 8p11↑(pt), 9q34↓(li), 10p11-pter↑(li), 10q21-q22↑(li), 10q26↓(li), 11q12-q13↑(pt), 14q32-qter↓(pt), 15q22-q23↓(li), 16p12↓(li), 17p12-p13↑(li), 17q12-q21↑(li), 18p11↑(li), 18q12-21↑(li), 20p11-p13↑(li), 20q13↑(li), 21q22-qter↓(li) |
| L23 | 1p36↓, 1q31-qter↑, 2q14-q21↑↑, 2q34-q37↓, 3p↓, 3q23-qter↑↑, 5p↑↑, 6p22-p24↓, 6p11-p21↑↑, 7q21↑, 8q12-qter↑, 10q21↓, 15q21-qter↑, 16p11-p12↓, 18p11.2↑, 18q21-22↑, 19q13↑↑, 20q12-q13↑ | 2p24-pter↓(pt), 4q24-qter↓(pt), 4q28-qter↑(pl), 5q12-q23↓(pt), 5q32-qter↑(pl), 7p11-p21↑(pl), 8p21-p22↓(pt), 9p13-pter↓(pt), 10q23-qter↓(pt), 11p14↓(pt), 12p11-p12↑(pl), 11q13-qter↓(pt), 13q21-qter↓(pt), 14q12↑(pl), 14q21-qter↓(pt), 17p11-p12↓(pt), 17p11-p13↑(pl), 19p11-pter↓(pl), 20p12↓(pt), 22q11-q12↑(pt) |
| L34 | 1p12-p31↓, 1q21↑, 2p↓, 2q31↑, 2q36-qter↓, 3p↓, 3q12-q25↑, 3q26-qter↓, 4↓, 5p↑, 5q↓, 6q11-q24↓, 7p11-p12↓, 7q21-qter↓, 8p↓, 8q↑, 9pter-q22↓, 9q31-ter↑, 10p14↑, 10p12-q24↓, 10q26↓, 11↑, 12pter-q15↑, 12q23-qter↑, 13q↓, 15q11-q15↑, 15q22-q24↑, 16p13↓, 16q23-qter↓, 17p↓, 18p↓, 18q↑, 19p↑, 20p12-pter↓, 21q↓ | 1q22-q41↑(pt,li2), 2q11-q24↓(li2), 6p↓(pt), 6p↑(li2), 6q25-qter(pt,li2), 7p13-pter↓(li2), 7q11↓(li2), 8q↑↑(li2), 14q24↓(pt), 14q11-q23↑(li2,li3), 16q11-q22↓(li2), 17q↑(pt), 17q↑(pt), 17q21↑(li1), 17q25↑(li1), 18q↑↑(li2), 20q11↑(pt), 20q13↑(pt), 20q↓(li2), 22↓(li2) |
| L27 | 1p13-p22↓, 1q23-q25↑, 1q32-q42↑, 2q21-q22↑, 3p11↓, 3q25-q28↑, 4q13-q28↓, 4q31↓, 4q34↓, 5q12-q22↓, 6p12-p21↑, 7q11↓, 7q22-q34↑, 8p22-p23↓, 9p12-p22↓, 10p13↑, 10q22-q23↓, 10q26↓, 11p11-p14↑, 11q14↑, 12q24.1↑, 13q34↓, 14q24↓, 15q24↑, 15q15↓, 16q13↑, 16q23-q24↓, 17q22↑, 19q13.1↑, 21q11-q22↓, 22q11-q13↑ | 1p32-p34↑(pt), 1p21-p22↑(ki), 1p32-p36↓(li), 1p34↓(ki), 1q31↑(li,ki), 1q43-qter↑(li,ki), 2p12-p24↑(li,ki), 2q23-q32↑(li,ki), 2q32-q36↑(ki), 3p12-pter↓(pt), 3p14-p21↓(ki), 3q21↑(ki), 3q12↑(ki), 3q25-q28↑↑(li) 4p12-pter↓(pt), 4p11-q11↓(li), 4q32↓(pt), 4q35↓(pt), 5p↑(li), 5q23-qter↓(pt), 5q35↓(ki), 6p11-p21↑↑(li), 6p22-pter↑(ki), 6q11-q22↑(ki), 7p11-p12↓(pt), 7q21↑(li,ki), 7q35-qter↑(li,ki), 8p11-p21↓(ki), 8q13-qter↑(ki), 8q24↑(li), 9p23-pter↓ (pt), 9q11-q12↓(li,ki), 9q22-q32↑(li,ki), 10p14-pter↑(ki), 10p12-q11↓(li), 10q21↓(pt), 10q24-q25↓(pt), 11q23-qter↓(pt), 12p11-pter↑(ki), 12q24.2-qter↑(li), 12q11-q12↓(li), 12q14-q23↑(ki), 13q11-q33↓(pt), 13q11-q12↓(ki), 13q21↑(li), 14q21↑(li,ki), 14q12↓(pt), 15q25↓(pt), 15q11-q14↓(ki), 15q21↓(ki), 16q21-q23↑(li), 16p11-p13↓(ki), 17p12↑(li), 17q11-q21↑(pt), 17q23-q25↑(li), 18p11↑(li,ki), 18q12-q23↑(ki), 19p11-p12↑(pt), 19q13.1↑↑(li), 19q13.2-13.3↑(pt), 20p12↑↑(li), 20q13.1↑(pt) |
| L39 | 1p31-p34.1↑, 1q21-q42↑, 2q23-q36↓, 3q27-qter↑, 4p12-p15.1↓, 4q22-q34↓, 5p↑, 5q↓, 6p↑, 6q11-q14↓, 7q21↓, 8p12-pter↓, 8q24↑, 9p↓, 9q21-q33↓, 10q↓, 11p↓, 11q13↑, 12q13-q14↑, 12q21-q23↓, 16q22↑, 18p11-q11↑, 18q12-qter↓, 20p11-qter↑ | 1p21-p31↓(li2), 3p↓(li1,li2), 3p14-p26↑(pt), 3q11-q25↓(li1,li2), 4q13-q21↓(li1,li2), 6q15-q23↓(li1,li2), 6q24-qter↓(li2), 7p13-pter↑(pt), 7p12-q21↓(li1), 7p11-p21↓(li2), 7q21↓(li2), 7q31↓(li2), 7q21-qter↓(pt), 8p11-q23↑(pt), 8q22-q23↑(li1), 8q12-q23↓(li2), 10p14-pter↑(pt), 11q24-q25↓(li1), 11q14-qter↓(li2), 13q↓(pt), 13q14-q33↓(li2), 14q24-qter↓(pt), 14q21-qter↓(li2), 14q11-q12↑(li1,li2), 15q11-q13↑(li1,li2), 15q23↑(pt,li2), 15q26↓(li2), 16p13-q21↑(li2), 16q11-q23↑(pt), 17p11-q21↑(li2), 17↑(pt), 19p11-p13↓(li1), 19p13-qter↑(li2), 19q13.1↑(li1), 20p12-pter↓(pt), 20p12↑((li1,li2), 21q21↓(pt,li2), 22q11-q13↑(li1,li2) |
| L40 | 1p11-p34↑, 1q11-q41↑, 2↑, 3p↓, 3q↑, 4q22-qter↓, 5q11-q23↓, 5q32-q33↑, 6p23-q13↑, 6q14-q21↑, 6q22-qter↓, 7↑, 9pter-q21↓, 9q22-q34↑, 10p12↑, 10q↓, 11p15↓, 11p13-p14↑, 11q12-q24↑, 12↑, 13q↓, 14q11-q31↓, 15q12-q21↑, 15q23-q25↑, 16p12-p13↑, 16q↓, 17p11↓, 17q21-q23↑, 19p12-p13↑, 19q13↑, 20↓, 22q↓ | 4p12-p15↑(ln1,ln2,ad), 4q13-q21↑(ln1,ln2,ad), 5p14↑(ln2,ad), 8↑(ln2,ad), 14q32↓(pt,ln2,ad), 17p12-pter↓(pt,ln1,ln2), 17q24-qter↑(pt,ln2,ad), 18q11-q22↑, 21q21↑(ad) |

pt = primary tumor; ln = lymph node metastasis; lu = intrapulmonary metastasis; ki = kidney metastasis; li = liver metastasis; p = pleura; ad = adrenal gland metastasis; om = omentum metastasis; ↓ = DNA loss; ↑ = DNA gain; ↑↑ = DNA amplification.

© 2000 Cancer Research Campaign

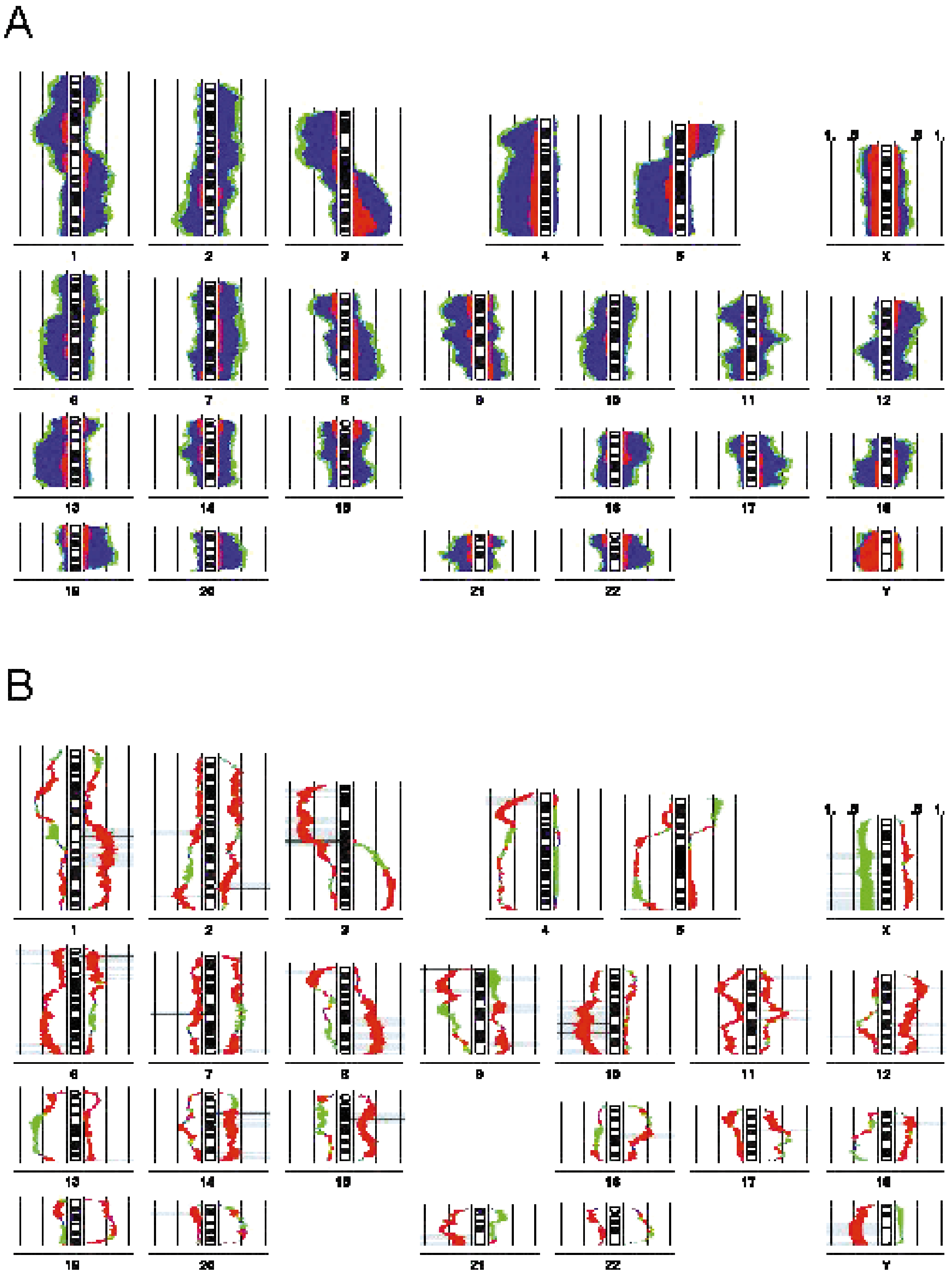

**Figure 1** (**A**) Summary of the chromosomal alterations in 50 SCCs of the lung in a histogram representation. The chromosomal imbalances are shown as incidence curves along each chromosome. Areas on the left side of the chromosome ideogram correspond to loss of genetic material; those on the right side correspond to DNA gains. The frequency of the alterations can be determined from the 0.5 (50%) and 1.0 (100%) incidence lines depicted parallel to the chromosome ideograms. DNA changes with 99% significance are coloured in blue, additional changes with 95% significance are depicted in green. The proportion of pronounced DNA gains and losses being defined as imbalances for which the ratio profiles exceeded the thresholds of 1.5 and 0.5, respectively, are visualized in red. They are most likely to represent high copy amplifications or multicopy deletions. The centromeric regions especially of chromosomes 1, 9, 13, 14, 15, 16, 19, 21 and 22, and the sex chromosomes must be excluded from the evaluation. (**B**) Difference histogram between non-metastatic and metastatic SCC of the lung. Green, percentage of changes that are exclusively present in non-metastatic SCC. Red, excess of changes in metastatic tumours. White areas beneath the coloured parts of each histogram, percentage of changes that are present in both tumour subgroups. Grey horizontal lines, statistically significant differences between non-metastatic and metastatic lung SCC. Light grey lines, regions with 95% significance; dark grey lines, 99% significance according to the $\chi^2$ test. Again, the centromeric regions must be excluded due to variability of fluorescence signals in these regions.

 *© 2000 Cancer Research Campaign*

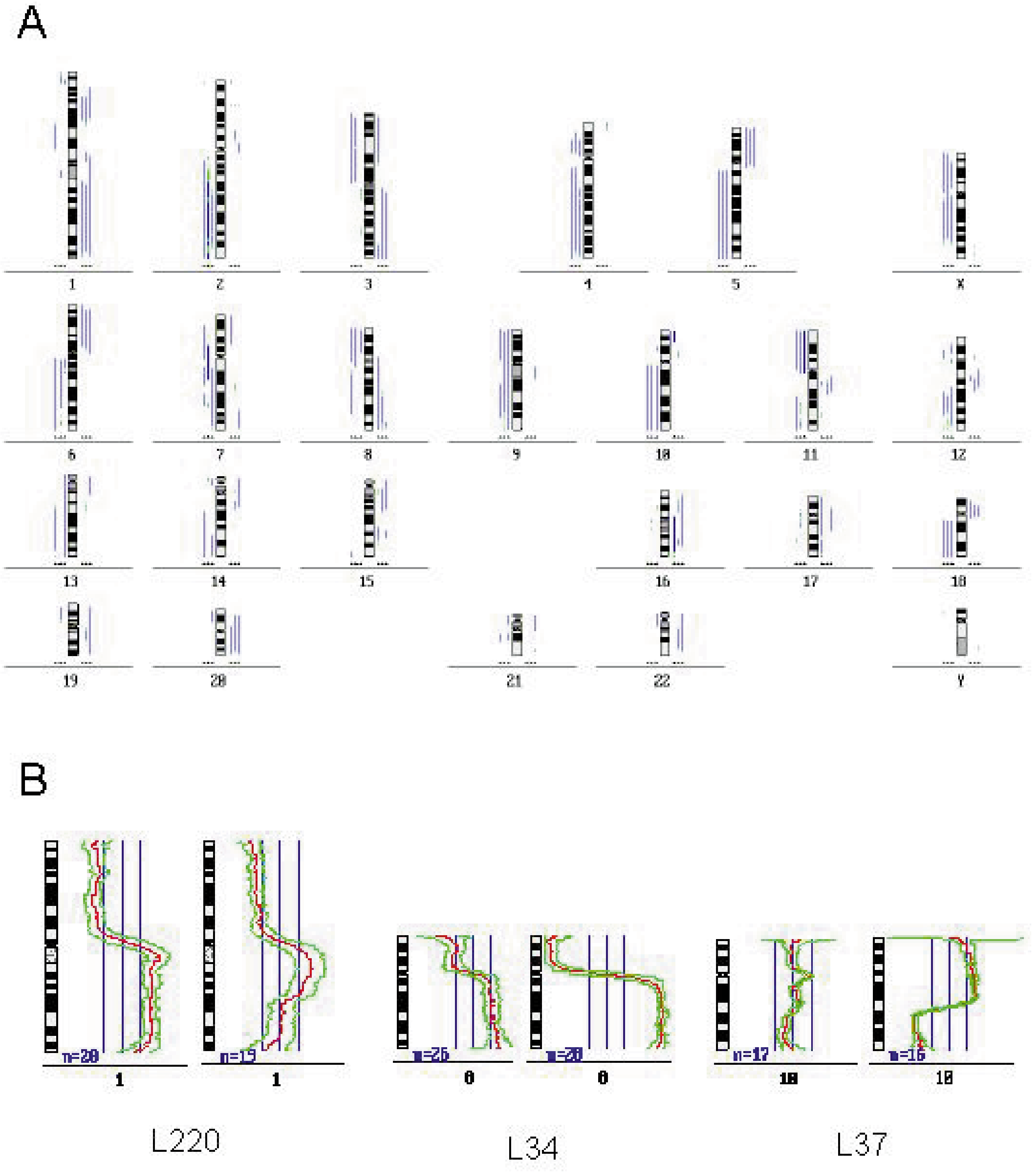

**Figure 2** (**A**) The CGH results of a primary tumour (case L39) and two of its liver metastases are shown by a line representation. Lines on the left side of the ideograms represent deletions, lines on the right side indicate DNA gains. The alterations of the primary tumour are depicted in the indexed position proximal to the chromosome ideogram, the lines in the middle position and the third position from the ideogram correspond to a large and small liver metastases respectively. The aberration pattern of chromosomes 4, 5, 6p, 8p, 9, 10q, 11p, 18 and 20 clearly indicates the clonal relationship between the three tumours. In addition, the comparison of the alterations of chromosomes 2q, 6q and 8 revealed that these deletions had expanded during tumour progression. (**B**) Examples of specific chromosome ratio profiles of three primary tumours (left) and their corresponding metastases (right). The profiles are shown along with the 99% confidence interval. The number of chromosomes included into the statistical analysis are indicated at each chromosome ideogram. The example of chromosome 1 suggested that the overrepresentation of the entire long arm was reduced to a DNA gain of the region 1q21–q25 most probably carrying the metastasis-associated gene. The example of chromosome 8 indicates the intensification of chromosomal change with the transition from low copy to high copy imbalances. The example of chromosome 10 suggests a triggering effect of small interstitial deletions.

© 2000 Cancer Research Campaign pronounced difference between the tumour groups. The differences were tested for significance by a $\chi^2$ test. Areas with 95% significance ($0.01 < P < 0.05$) are depicted in bright grey, and areas with 99% significance ($P < 0.01$) are depicted in dark grey.

Detailed protocols of the preparation and digital image analysis have been published previously (Petersen et al, 1997*a* , 1997*b*; Roth et al, 1997) and are available on our website *http://amba.charite.de/cgh*.

### RESULTS

The chromosomal imbalances of 50 lung SCCs are summarized in Figure 1A by a histogram representation. One tumour per patient was included in the histogram. The most prevalent deletions with an incidence of more than 50% were observed for chromosomes 1p21–p31, 2q34–q36, 3p, 4p, 4q, 5q, 6q14–q24, 8p, 9p, 10q, 11p12–p14, 13q13–qter, 18q12–qter and 21q21. In addition, the following regions were slightly less frequently affected by deletions (30% < incidence ≤ 50%): 1p34–p36, 2q22–q33, 2q37, 9q13–q31, 10p, 11q14–qter, 12q21, 14q21, 15q21 and 17p.

Most prevalent DNA over-representations (> 50%) were seen on chromosomes 1q11–q25, 1q32–q41, 3q, 5p, 8q22–qter, 11q13, 12p, 17q21–q22, 17q24–q25, 19, 20q and 22q and less frequently (30% < incidence ≤ 50%) on 1p32–pter, 2pter–2q21, 2q24–q32, 6p11–p21, 7, 8q11–q21, 9q22–q34, 12q23–q24.3, 14, 15q11–q14, 15q23–q25, 16p and 18p11.2–q11.2.

Pronounced DNA copy number changes generally co-localized with peaks of the incidence curve, i.e. deletions at 1p22–p31, 3p12–p14, 4p14–p15.1, 8p12–p22, 9p21, 10q21, 10q23, 11p13–p14, 11q14–q22, 11q24–q25, 13q21–q31, 14q21, 15q21, 18q12–q21, 18q22 and 21q21, and over-representations at 1q21–q25, 1q32–q41, 2q31, 3q27–q28, 5p15.3, 6p12, 7p21–p22, 7p12–p13, 7q21–q22, 8q13–q21.1, 8q22, 8q24.1, 9q34, 11q13, 12p, 12q13, 13q33–q34, 14q23–q24, 17q21–q22, 17q24–q25, 18q11, 19p13.3, 19q13–1. q13.2, 20p12, 20q and 22q.

In general, lung SCC showed a very complex pattern of DNA imbalances in which almost any chromosome region was affected by DNA gains and losses. The only exception was the over-representation of chromosome 3p that was never observed even by our quite sensitive statistical method for the determination of copy number changes.

The direct comparison of ten primary tumours and their corresponding metastases is summarized in Table 1. It revealed a large number of common changes between the tumours of the same patient, indicating a clonal relationship as well as a considerable number of aberrant alterations. Thus, the interpretation of this data is complicated by the considerable genetic instability and heterogeneity of lung carcinomas. Therefore we decided to focus on the comparison of tumour groups, i.e. metastatic and non-metastatic lung SCC, since it facilitates the identification of those imbalances relevant for the metastatic phenotype by reducing the influence of tumour heterogeneity.

The statistical analysis of the metastatic and non-metastatic SCC by a difference histogram is represented in Figure 1B. It is based on the comparison of 25 primary tumours without evidence of metastases formation at the time of diagnosis, i.e. of stage pN0/M0, and 25 metastasizing carcinomas. Only few chromosomal regions carry an excess of changes in the non-metastatic subgroup represented by the green colour when compared to the metastatic carcinomas. One such region was observed on chromosome 5p which, however, failed to achieve statistical significance. The overall excess of changes in the metastatic tumour group is visible by the prevailing red colour in the difference histogram.

For the most prevalent imbalance region (incidence > 50%), the statistical comparison using the $\chi^2$ test indicated that DNA losses of the chromosome regions 3p12–p14, 3p21, 4p15–p16, 8p22–p23 and 10q21–qter, as well as the over-representations of the chromosomal bands 1q21–q25 and several regions on 8q, were associated with a statistical significance for the metastatic phenotype. For the less frequent changes (30% < incidence ≤ 50%), the statistical analysis indicated in addition deletions on 6q24–qter and 21q22, and the DNA gains at 9q34, 14q12 and 15q12–q15. These chromosomal regions were consistently found when different evaluation schemes, i.e. using fixed ratio thresholds and statistical methods, were applied for the determination of the imbalances of individual cases. These different evaluation schemes are accessible at our web site http://amba.charite.de/cgh. For single chromosome regions, there was, however, a certain variability for locus assignment.

The direct comparison of ten primary tumours and their corresponding metastases confirmed the results of the statistical comparison of the tumour subgroups since single cases showed the statistically significant changes additionally in the metastatic lesions. In the majority of cases, however, these changes were already detectable in the primary tumour. In addition, the comparison suggested distinct genetic changes in the evolution of chromosomal imbalances during tumour progression. Typical examples of the comparison are shown in Figure 2. The CGH analysis of a primary tumour and two liver metastases is shown in Figure 2A. One metastasis measured 2.5 cm and the other 1 cm in diameter. The profiles for chromosomes 2q, 6q and 8 indicated that size of deletions increased during tumour progression. The deletion on chromosome 3p including the band 3p12 was not detectable in the primary tumour.

In Figure 2B, examples of specific chromosome ratio profiles of three primary tumours and one of their corresponding metastases is shown. The example of chromosome 1 indicated that the over-representation of the entire long arm was reduced to a DNA gain of the region 1q21–q25, which is the region that was also indicated by the statistical comparison of the tumour subgroups thus most probably carrying the metastasis-associated gene. We observed an identical transition twice exclusively for chromosome 1q. The example of chromosome 8 indicates the intensification of chromosomal change with the transition from low copy to high copy imbalances. The example of chromosome 10 suggests a triggering effect of small imbalances. Again it illustrates the extension of deletions during tumour progression, which was a general finding and does not seem to be restricted to specific chromosomes. The detailed comparison of the primary tumours and their metastases is listed in Table 1. The profiles and line representations of all ten cases can also be accessed at our web page *http://amba.charite.de/cgh.*

### DISCUSSION

In the present study we used CGH to define the frequency of chromosomal imbalances in a collective of lung SCC and to search for genetic alterations that are associated with the metastatic phenotype.

© 2000 Cancer Research Campaign

### CGH histograms of lung SCC

The histogram of 50 lung SCCs indicates the incidence of DNA gains and losses at specific chromosome sites providing an overview of those regions that are characteristically affected in this tumour entity. We applied a quite sensitive method for the determination of chromosomal imbalances. It is based on the statistical evaluation whether the deviation of the ratio profile from the normal ratio value 1.0 is significant or not. In contrast to the evaluation by fixed thresholds, no specific ratio values were defined to score a DNA gain or loss. The comparison in sensitivity can be seen by the profile of tumour L37 in Figure 2B. Since the profile with its confidence interval of the primary tumour shows a deviation from the normal ratio value 1.0, DNA imbalances are scored by the statistical method, whereas no change would be detected by using the ratio thresholds 0.75 and 1.25 (e.g. Joos et al, 1995), or even the more sensitive values of 0.85 and 1.15 (e.g. Tarkkanen et al, 1995). The threshold-based methods used in the initial period of CGH, which are still very popular, are probably partially responsible for the higher frequency of DNA gains compared to DNA losses that have been detected in many studies. Although statistical evaluation is evolving as the method of choice (Moore et al, 1997; Kirchhoff et al, 1998), there is not yet a consensus about the best way to determine DNA changes by CGH.

The higher sensitivity of the statistical method does correlate to the relatively high incidence of imbalances observed in this study. Although the exact frequency still needs to be determined, the histogram does visualize the most frequently affected sites and their relative importance. Another advantage of the histogram representation is the fact that it enables the statistical comparison of tumour subgroups. To our knowledge it provides the first method to search genome wide for phenotype–genotype correlations (Petersen et al, 1997*b*). Again, for this comparison it is important that DNA gains and losses are detected with equal sensitivity. The good correlation of the result of the difference histogram with our allelotyping analysis, which was performed on the similar tumour collective, supports the validity of our CGH findings (Petersen et al, 1998*a*, 1998*b*). We identified several candidate regions associated with metastasis formation and tumour progression. Although the exact localization of corresponding genetic defects needs to be confirmed by future studies we think that the overall complexity of the CGH pattern corresponds well with the intricacy of systemic tumour dissemination.

### Chromosomal alterations associated with the metastatic phenotype

The difference histogram suggested that the deletions of several chromosomal regions were statistically significant for the metastatic phenotype, e.g. deletions at 3p12–p14, 3p21, 4p15–p16, 6q24–qter, 8p22–p23, 10q21–qter and 21q22. Some of these regions have already been implicated in tumour progression and metastases formation in other neoplasms, supporting the hypothesis that many features of tumour cell dissemination are not specific for one entity but are shared between multiple tumour types.

Deletions of the chromosome band 4p16 have recently been observed more frequently in metastases of colorectal carcinomas than in primary tumours (Paredes-Zaglul et al, 1998). In SCLC, Levin et al observed a metastatic lesion with a deletion of chromosome 4p that was not detectable in the primary tumour (Levin et al, 1995). For chromosomes 6q, 8p and 10q there is functional as well as genetic evidence suggesting that they harbour genes that are important in metastases formation. Chromosome transfer experiments revealed that they were able to suppress the metastatic potential of either rodent or human cell lines (Welch et al, 1994; Ichikawa et al, 1996). Three regions of allelic loss on chromosome 8p have been defined, particularly in high grade head and neck SCC (Wu et al, 1997). Deletions of chromosome 10q have been described in multiple advanced solid tumours. We previously showed that loss of heterozygosity (LOH) was significantly more frequent in metastatic tumours and defined three distinct regions of allelic imbalance. Meanwhile, several candidate genes have been identified most of which, however, seem to be of minor importance in lung cancer (Petersen et al, 1998*a*, 1998*b*).

Regarding chromosome 21q there have been only limited data that it is involved in tumour progression. Deletions on 21q were recently found to be statistically more significant in brain metastases of NSCLC than in early stage primary tumours (Kohno et al, 1998).

Chromosome 3p has been implicated particularly in tumour initiation (Hung et al, 1995). Interestingly, our data suggested that deletions of 3p12–p14 are important for cancer progression. Whereas non-metastatic tumours more frequently carry interstitial deletions, the aggressive carcinomas showed the loss of the entire chromosome arm including the band 3p12–p14. This is a typical finding in SCLC (Petersen et al, 1997*a*).

In general, the analysis by the difference histogram strengthens the notion that the accumulation of deletions with the putative inactivation of tumour suppressor genes is particularly important for haematogeneous tumour spread. However, there were also some DNA over-representations associated with the metastatic phenotype, e.g. gains of 1q21–q25, 8q, 9q34, 14q12 and 15q12–q15.

The over-representation on chromosome 1q21–25 being of 95% significance has been previously reported in metastatic renal cell carcinomas (Gronwald et al, 1997). DNA gains on 8q, and in particular amplifications of the c-myc proto-oncogene at 8q24, are genetic markers for tumour progression (Brison et al, 1993). Recently the 8q gain along with the 8p loss has been found in bone metastases of prostate cancer (Alers et al, 1997). To our knowledge the gains at 9q34 and 15q11–q13 have not yet been reported in advanced tumour stages. The chromosomal band 9q34 as well as chromosomes 19, 22q and the very telomeric regions, e.g. 8q24, might give rise to false positive results in CGH analysis (Moore et al, 1997; Kirchhoff et al, 1998). Nevertheless, we feel that these over-representations are valuable findings since we also observed high copy over-representations of these regions which can not be simply regarded as CGH artefacts. The Abe gene is one candidate for the 9q34 region. For chromosome 15q, only allelic loss has been so far observed in association with the metastatic phenotype of breast carcinomas (Wick et al, 1996).

### Chromosomal alterations in the clonal evolution of metastatic tumour cells

In general, the observation that metastatic lung SCCs carry more chromosomal alterations than the pM0 tumours supports the hypothesis that they are derived from non-metastatic precursors by the acquisition of additional changes, suggesting a clonal evolution. This clonal relationship was confirmed in each of the ten cases

*© 2000 Cancer Research Campaign*

in which multiple tumours were investigated similar to our previous study in SCLC (Schwendel et al, 1997). However, it is in contrast to CGH studies on other tumour entities in which clonality could not be established in every case (Kuukasjarvi et al, 1997). This seeming discrepancy can be explained by several factors. First, tumour heterogeneity might heavily influence the establishment of genetic correlations especially if there is a long latency period for the occurrence of metastases. Second, normal cell contamination and the quality of tumour DNA may reduce the sensitivity for the detection of chromosomal imbalances by CGH (Isola et al, 1994). We investigated exclusively synchronous metastases and DNA from frozen tumour tissue that was controlled for the amount of contaminating normal tissue in each case.

The comparison of primary tumours and their corresponding metastases confirmed the findings of the statistical analysis by the difference histogram in single cases. Additionally it suggested distinct genetic changes during the process of tumour progression being exemplified in Figure 2. First, for chromosome 1q we observed twice over-representations of the entire chromosome arm in the primary tumours, whereas the metastases showed DNA gain or even amplification of single bands. This correlates with the finding that gene amplifications are typically found in advanced tumour stages (Brison et al, 1993). Second, as a more general finding we observed that small interstitial deletions were often extended to large DNA under-representations that affected chromosome arms or even entire chromosomes. This observation points to the importance of triggering effects in the evolution of chromosomal defects. Third, the genetic changes were often more pronounced in the metastatic lesion. This might be explained by the fact that out of the genetically heterogeneous primaries those cells which carry a selection advantage for tumour dissemination are enriched in the metastatic lesions. In addition, the metastatic lesions do generally have a less pronounced contamination by normal tissue.

In summary, we identified several chromosomal imbalances that were associated with the metastatic phenotype of lung SCC, offering new candidate regions for the identification of metastasis-associated genes. Importantly, the specific chromosome defects were frequently detectable in the primary tumours thus offering a mean to evaluate the patient risk by the analysis of biopsies or tumour resections. Although these changes were generally not simultaneously detectable in a single case it is obviously the accumulation that mediates the potential for tumour dissemination. The complexity of the genetic changes already detectable by CGH clearly indicates that it will be necessary to monitor a large number of genes to evaluate the malignant potential of a tumour. The identification of these genes and their role in tumour progression will be a fascinating task in the forthcoming years. Despite the limitation of CGH being the fact that it does not detect defects of specific genes its power to provide a comprehensive picture of the chromosomal alterations has revealed a remarkable phenotype–genotype correlation in lung carcinomas. Therefore CGH does supply valuable information for a refined tumour characterization and may serve as a paradigm for the feasibility of a genetic classification of solid tumours.

### ACKNOWLEDGEMENTS

The technical assistance of Nicole Deutschmann is gratefully acknowledged. The study was supported by the DFG grant Pe 602/1.

### REFERENCES

Alers JC, Krijtenburg PJ, Rosenberg C, Hop WC, Verkerk AM, Schroder FH, van der Kwast TH, Bosman FT and van Dekken H (1997) Interphase cytogenetics of prostatic tumor progression: specific chromosomal abnormalities are involved in metastasis to the bone. *Lab Invest* **77**: 437–448

Bockmühl U, Petersen S, Schmidt S, Wolf G, Jahnke V, Dietel M and Petersen I (1997) Patterns of chromosomal alterations in metastasizing and nonmetastasizing primary head and neck carcinomas. *Cancer Res* **57**: 5213–5216

Brison O (1993) Gene amplification and tumor progression. *Biochim Biophys Acta* **1155**: 25–41

Gronwald J, Storkel S, Holtgreve-Grez H, Hadaczek P, Brinkschmidt C, Jauch A, Lubinski J and Cremer T (1997) Comparison of DNA gains and losses in primary renal clear cell carcinomas and metastatic sites: importance of 1q and 3p copy number changes in metastatic events. *Cancer Res* **57**: 481–487

Hung J, Kishimoto Y, Sugio K, Virmani A, McIntire DD, Minna JD and Gazdar AF (1995) Allele-specific chromosome 3p deletions occur at an early stage in the pathogenesis of lung carcinoma. *JAMA* **273**: 558–563

Ichikawa T, Nihei N, Kuramochi H, Kawana Y, Killary AM, Rinker-Schaeffer CW, Barrett C, Isaacs JT, Kugoh H, Oshimura M and Shimazaki J (1996) Metastasis suppressor genes for prostate cancer. *Prostate Suppl* **6**: 31–35

Isola J, de Vries S, Chu L, Ghazvini S and Waldman FM (1994) Analysis of changes in DNA sequence copy number by comparative genomic hybridization in archival paraffin embedded tumor samples. *Am J Pathol* **145**: 1301–1308

Joos S, Bergerheim US, Pan Y, Matsuyama H, Bentz M, du Manoir S and Lichter P (1995) Mapping of chromosomal gains and losses in prostate cancer by comparative genomic hybridization. *Genes Chromosomes Cancer* **14**: 267–276

Kirchhoff M, Gerdes T, Rose H, Maahr J, Ottesen AM and Lundsteen C (1998) Detection of chromosomal gains and losses in comparative genomic hybridization analysis based on standard reference intervals. *Cytometry* **31**: 163–173

Kohno T, Kawanishi M, Matsuda S, Ichikawa H, Takada M, Ohki M, Yamamoto T and Yokota J (1998) Homozygous deletion and frequent allelic loss of the 21q11.1–q21.1 region including the ANA gene in human lung carcinoma. *Genes Chromosomes Cancer* **21**: 236–243

Kuukasjarvi T, Karhu R, Tanner M, Kahkonen M, Schaffer A, Nupponen N, Pennanen S, Kallioniemi A, Kallioniemi OP and Isola J (1997) Genetic heterogeneity and clonal evolution underlying development of asynchronous metastasis in human breast cancer. *Cancer Res* **57**: 1597–1604

Levin NA, Brzoska P, Gupta N, Minna JD, Gray JW and Christman MF (1994) Identification of frequent novel genetic alterations in small cell lung carcinoma. *Cancer Res* **54**: 5086–5091

Levin NA, Brzoska PM, Warnock ML, Gray JW and Christman MF (1995) Identification of novel regions of altered DNA copy number in small cell lung tumors. *Genes Chromosomes Cancer* **13**: 175–185

Moore DH II, Pallavicini M, Cher ML and Gray JW (1997) A t-statistic for objective interpretation of comparative genomic hybridization (CGH) profiles. *Cytometry* **28**: 183–190

Paredes-Zaglul A, Kang JJ, Essig YP, Mao W, Irby R, Wloch M and Yeatman TJ (1998) Analysis of colorectal cancer by comparative genomic hybridization: evidence for induction of the metastatic phenotype by loss of tumor suppressor genes. *Clin Cancer Res* **4**: 879–886

Petersen I, Langreck H, Wolf G, Schwendel A, Psille R, Vogt P, Reichel MB, Ried T and Dietel M (1997*a*) Small cell lung cancer is characterized by a high incidence of deletions on chromosomes 3p, 4q, 5q, 10q, 13q and 17p. *Br J Cancer* **75**: 79–86

Petersen I, Bujard M, Petersen S, Wolf G, Goeze A, Schwendel A, Langreck H, Gellert K, Reichel M, Just K, du Manoir S, Cremer T, Dietel M and Ried T (1997*b*) Patterns of chromosomal imbalances in adenocarcinoma and squamous cell carcinoma of the lung. *Cancer Res* **57**: 2331–2335

Petersen S, Wolf G, Bockmühl U, Gellert K, Dietel M and Petersen I (1998*a*) Allelic loss on chromosome 10q in human lung cancer: Association with tumor progression and metastatic phenotype. *Br J Cancer* **77**: 270–276

Petersen S, Rudolf J, Bockmühl U, Gellert K, Wolf G, Dietel M and Petersen I (1998*b*) Distinct regions of allelic imbalance on chromosome 10q22–q26 in squamous cell carcinomas of the lung. *Oncogene* **17**: 449–454

Pisani P, Parkin DM and Ferlay J (1993) Estimates of the worldwide mortality from eighteen major cancers in 1985. Implications for prevention and projections of future burden. *Int J Cancer* **55**: 891–903

Ried T, Petersen I, Holtgreve-Grez H, Speicher MR, Schröck E, du Manoir S and Cremer T (1994) Mapping of multiple DNA gains and losses in primary small cell lung carcinomas by comparative genomic hybridization. *Cancer Res* **54**: 1801–1806

 © 2000 Cancer Research Campaign

Roth K, Wolf G, Dietel M and Petersen I (1997) Image analysis for comparative genomic hybridization based on a karyotyping program for Windows. *Anal Quant Cytol Histol* **19**: 461–474
Schwendel A, Langreck H, Reichel M, Schröck E, Ried T, Dietel M and Petersen I (1997) Primary small cell lung carcinomas and their metastases are characterized by a recurrent pattern of genetic alterations. *Int J Cancer (Pred Oncol)* **74**: 86–93
Sekido Y, Fong KM and Minna JD (1998) Progress in understanding the molecular pathogenesis of human lung cancer. *Biochim Biophys Acta* **1378**: F21–F59
Tarkkanen M, Karhu R, Kallioniemi A, Elomaa I, Kivioja AH, Nevalainen J, Bohling T, Karaharju E, Hyytinen E and Knuutila S (1995) Gains and losses of DNA sequences in osteosarcomas by comparative genomic hybridization. *Cancer Res* **55**: 1334–1338
Welch DR, Chen P, Miele ME, McGary CT, Bower JM, Weissman BE and Stanbridge EJ (1994) Microcell-mediated transfer of chromosome 6 into metastatic human C8161 melanoma cells suppresses metastasis but does not inhibit tumorigenicity. *Oncogene* **9**: 255–262
WHO (1981) Histological typing of lung tumors. WHO: Geneva
Wick W, Petersen I, Schmutzler RK, Wolfarth B, Lenartz D, Bierhoff E, Hümmerich J, Müller DJ, Stangl AP, Schramm J, Wiestler OD and von Deimling A (1996) Evidence for a novel tumor suppressor gene on chromosome 15 associated with progression to a metastatic stage in breast cancer. *Oncogene* **12**: 973–978
Wu CL, Roz L, Sloan P, Read AP, Holland S, Porter S, Scully C, Speight PM and Thakker N (1997) Deletion mapping defines three discrete areas of allelic imbalance on chromosome arm 8p in oral and oropharyngeal squamous cell carcinomas. *Genes Chromosomes Cancer* **20**: 347–353

*© 2000 Cancer Research Campaign*
